# Analyzing the Effect of Arbuscular Mycorrhizal Fungi and Plant Growth-Promoting Bacteria Inoculation over the Growth of Tomatoes in a Martian Regolith Analog: Perspectives for Martian Agriculture †

**DOI:** 10.3390/microorganisms14010200

**Published:** 2026-01-15

**Authors:** Daniel Fernando Cortez Acosta, Víctor Olalde Portugal, Rufino Lozano Santacruz, Sergio Valle Cervantes

**Affiliations:** 1 Departamento de Biotecnología y Bioquímica, Centro de Investigación y Estudios Avanzados del Instituto Politécnico Nacional, Unidad Irapuato, Irapuato 36824, Mexico; 2 Laboratorio Nacional de Geoquímica y Minerología (LANGEM), Instituto de Geología, Universidad Nacional Autónoma de México, Ciudad Universitaria, Alcaldía Coyoacán, Ciudad de México 04510, Mexico; rufino@unam.mx; 3 Departamento de Ingenierías Química y Bioquímica, Maestría en Sistemas Ambientales, Instituto Tecnológico de Durango, Durango 34080, Mexico; svalle@itdurango.edu.mx

**Keywords:** Mars, perchlorate, Martian Regolith Analog, arbuscular mycorrhizal fungi, plant growth promoting bacteria

## Abstract

For future Mars colonization, crop production will be a challenge due to the chemical composition of the Martian Regolith, which contains perchlorates and heavy metals. This research was conducted to determine if the use of Arbuscular Mycorrhizal Fungi (AMF), Plant Growth-Promoting Bacteria (PGPB), and fertilization have a positive effect on tomato growth in a Martian Regolith Analog. The analog contains 52.54% SiO_2_, 1.81% TiO_2_, 17.66% Al_2_O_3_, 9.46% Fe_2_O_3_, 0.145% MnO, 3.43% MgO, 7.09% CaO, 3.95% Na_2_O, 1.96% K_2_O, and 0.55% P_2_O_5_. Two hundred and forty tomato plants were grown for 45 days. One hundred and twenty tomato plants grown over perchlorate-polluted analog (1% m/m) died in less than 2 weeks, while 120 tomato plants grown in a non-polluted analog survived. Forty-eight plants supplemented with Long–Ashton solution increased their shoot length 100% more than the control plants and the plants inoculated with the commercial AMF formulation TM-73^MR^ and PBB; the latter showed 25% mycorrhizal colonization. There was no significant difference between the growth parameters of inoculated plants and non-inoculated plants. However, there was a significant difference compared to the plants supplemented with Long–Ashton solution. The perchlorate is toxic to tomato plants, and the metal content of the analog was not a limiting factor for tomato growth or AMF colonization.

## 1. Introduction

One of the primary challenges for the future colonization of Mars is producing enough food for long-term human colonies. There are various approaches to solving this problem, such as hydroponics, aeroponics, or conventional agriculture using the Martian Regolith as a substrate for crop cultivation [1,2]. In the long term, conventional agriculture is likely to be the most cost-effective approach; however, the primary challenge with the Martian Regolith is its chemical composition. It contains large amounts of heavy metals, e.g., iron, and nutrients such as phosphorus (P) and potassium (K) are present in their oxidized forms, and thus, are not not bioavailable [3]. The most important limiting factor, however, is that the Martian Regolith contains 0.5–1% *w*/*w* perchlorates, which are toxic to many organisms, including humans [4] and plants [5]. In this scenario, it is crucial to develop strategies to enhance the availability of bioavailable nutrients in the Martian Regolith for future agricultural purposes. One interesting approach to solving this problem is to utilize microorganisms for bioaugmentation and biofertilization in the regolith using Plant Growth-Promoting Bacteria (PGPB) and Arbuscular Mycorrhizal Fungi (AMF). These microorganisms can leach nutrients (such as phosphorus) from the substrates, and they can be used to alleviate abiotic stress in plants caused by chemicals in the substrate, employing strategies such as promoting phytohormone production, siderophore production, and 1-aminocyclopropane-1-carboxylic acid (ACC) deaminase production [6,7].

To study that on Earth, Martian Regolith Analogs and Simulants (Earth soils with chemical and physical properties that resemble the real Martian Regolith) are used. In the past, some studies confirmed that bacterial genera such as *Bacillus* and *Enterococcus* were resistant to desiccation, perchlorate exposure, reduced atmospheric pressure, and UV exposure [8]. Also, the use of genera such as *Azotobacter*, *Pseudomonas*, *Methylobacterium*, and *Kosakonia* improved lettuce development in Martian and Moon Simulants [9]. Unfortunately, at this time, only reliable data on the Mojave Mars Simulant and Johnson Space Simulant are available [10], and the research is limited to a few countries because the Simulants and Analogs are expensive and not available in emerging countries. It is essential to find suitable analogs with higher availability to study the inoculation of microorganisms and their interactions with plants. In this context, the present research analyzed soil from an iron mine to determine its viability as a Martian Regolith Analog and designed an experiment to investigate whether tomato plants can grow in it. Additionally, we supplemented the plants with Arbuscular Mycorrhizal Fungi (AMF) and Plant Growth-Promoting Bacteria (PGPB) to evaluate whether this strategy could address nutrient limitations in the Martian Regolith and the presence of perchlorate.

## 2. Materials and Methods

### 2.1. Analog Selection and Characterization

Soil samples were obtained from “La Tortuga,” an iron mine located in Valle de Santiago, Guanajuato, Mexico (20.359366, −101.193120). A sample of approximately 40 kg of red soil (loamy sand, Horizon A) was collected and mixed using a mechanical mixer. To evaluate its suitability as a Martian Regolith Analog, we determined the chemical composition, potential of hydrogen (pH), and organic matter content using the following methods: X-ray fluorescence for chemical composition, the Food and Agriculture Organization (FAO) Standard operating procedure for soil pH determination [11] and the FAO Standard operating procedure for soil organic carbon [12]. X-ray fluorescence analysis to determine the soil’s chemical composition was conducted at the Laboratorio Nacional de Geoquímica y Minerología of the Universidad Nacional Autónoma de México (Ciudad de México, Mexico). Six random soil samples were milled into a powder (−200 mesh). We measured the concentrations of Silicon Dioxide (SiO_2_), Titanium Dioxide (TiO_2_), Aluminum Trioxide (Al_2_O_3_), Iron Trioxide (Fe_2_O_3_), Manganese Oxide (MnO), Magnesium Oxide (MgO), Calcium Oxide (CaO), Sodium Oxide (Na_2_O), Potassium Oxide (K_2_O), and Phosphorus Pentoxide (P_2_O_5_) in triplicate for each sample using a Rigaku sequential X-ray spectrometer (Rigaku, Tokyo, Japan) equipped with a rhodium radiation tube. The pH was measured using a Denver Instrument Basic pH Meter with a glass electrode (Denver Instrument Company, Arvada, CO, USA). Initially, soil samples were milled to achieve a particle size of less than 2 mm. The samples were then dried in an oven at 40 °C for 24 h and stored in a desiccator for a few minutes before measurement. The potentiometer was calibrated using three standard buffers (4.01, 7.00, and 10.00) before each determination. The dried soil was suspended in a 0.01 M Calcium Chloride (CaCl_2_) solution in 50 mL flasks (1 g of soil per 5 mL of solution) and mixed for 60 min on a mechanical shaker at 200 RPM. After allowing the mixture to stand for another 60 min, the pH electrode was placed in each flask to measure the pH level. Three random soil samples were analyzed in triplicate to obtain the final pH value [11]. The organic matter content was determined using the Walkley–Black colorimetric method. A standard curve using sucrose as the reference material was prepared, consisting of nine points, with two samples per point, each recorded three times (ranging from 0 mg to 8 mg of organic carbon). For each sample, 0.5 g of soil was placed in a tube, followed by the addition of 2 mL of a 0.34 M Potassium Dichromate (K_2_Cr_2_O_7_) solution. The mixture was stirred before adding 5 mL of Sulfuric Acid (H_2_SO_4_), and the solution was remixed after 30 min. 20 mL of distilled water was added to each tube, and the mixture was stirred again, then left to stand overnight. Finally, the absorbance of each sample was measured at 600 nm using a Thermo Fisher Plate Reader (Thermo Fisher Scientific, Waltham, MA, USA) [12]. Three random soil samples were analyzed in triplicate to determine the final organic matter content.

### 2.2. Perchlorate-Tolerant Microorganisms Isolation

An abstract of this section is available in “Revista de la Sociedad Mexicana de Biotecnología y Bioingeniería” in the Special Issue “Memorias del Congreso Nacional de Biotecnología y Bioingeniería 2023”. Soil samples were taken from “La Tortuga”, an iron mine located in Valle de Santiago, Guanajuato, Mexico (20.359366, −101.193120), and from arable soil at CINVESTAV, Irapuato, Guanajuato, Mexico (20.72120, −101.32792). The next methodology was used to isolate microorganisms:

• Soil Extract Agar (SEA) Preparation.—400 g subsample of each soil was suspended with 1 L distilled water, and sterilized at 120 °C for 1 h; then, the supernatant was recovered, adjusted to a pH of 7.0, and supplemented with 15 g/L agar; this formulation was sterilized again at 120 °C for 15 min and plated. (https://www.dsmz.de/microorganisms/medium/pdf/DSMZ_Medium12.pdf (accessed on 1 May 2022)).
• Microorganisms extraction from soils.—2 g soil samples were added to 2 mL of Phosphate Buffer (PB) in sterile centrifugal tubes, the solution was mixed, and then plated onto the SEA. The SEA was incubated at 30 °C for 3 days [13].
• Obtention of perchlorate-tolerant microorganisms.—The obtained microorganisms present in the SEA were transferred to Perchlorate Acetate Agar (PAG). This media were prepared according to the growth media described by [14], with some modifications. The media contained, per liter: 4.0 g sodium acetate (C_2_H_3_NaO_2_), 0.4 g ammonium phosphate (N_3_H_12_PO_4_), 0.2 g potassium phosphate dibasic (K_2_HPO_4_), 0.02 g magnesium sulfate (MgSO_4_·7H_2_O), 0.02 g sodium chloride (NaCl), 2 mg zinc sulfate (ZnSO_4_), 1 mg manganese chloride (MnCl_2_), 1 mg calcium chloride (CaCl_2_), 0.2 mg cupric sulfate (CuSO_4_), 0.4 mg cobalt chloride (CoCl_2_), 0.025 g sodium molybdate (Na_2_MoO_4_·2H_2_O), and 10 g of magnesium perchlorate Mg(ClO_4_)_2_. All the reagents were ACS-grade. The PAG was incubated at 30 °C for 7 days.
• Isolation of perchlorate-tolerant microorganisms.—The microorganisms that survived in PAG were isolated using the streak plate method [15] on the same media. The plates were incubated for 7 days at 30 °C and the process was repeated until individual colonies were isolated. To preserve all the isolated microorganisms, we reinoculated them on fresh PAG every month and stored the plates at 4 °C.

The beneficial traits we sought were phosphorus solubilization, potassium solubilization, siderophore production, and nitrogen fixation. The phosphate solubilization was determined using a modified Pikovskaya agar (https://www.himedialabs.com/media/TD/M520.pdf (accessed on 1 May 2022) and [16]), supplemented with 0.1 g/L of bromothymol blue to enhance the visibility of the solubilization halos. The isolated strains were then inoculated onto plates using sterile toothpicks to create spots, and the plates were incubated for 5 days at 30 °C. The potassium solubilization capacity was determined by growing the strains in Aleksandrow media (https://www.himedialabs.com/media/TD/M1996.pdf (accessed on 1 May 2022)) supplemented with 15 g/L of bacteriologic agar and 0.1 g/L of bromothymol blue solution to enhance halo visibility [17]. The nitrogen fixation test was conducted by growing microorganisms in tubes containing modified Nitrogen-Free Basal (NFB) semi-solid media [18] and observing their growth and color changes in the media. Finally, siderophore production was determined by growing microorganisms on Chrome Azurol S (CAS) blue agar [19] and observing color changes. All plates and tubes were incubated at 30 °C for 5 days, and color changes in the media were used as criteria to determine positive results.

The strains that showed phosphate solubilization, potassium solubilization, nitrogen fixation, and siderophore production were identified by two methods: 16s Ribosomal Deoxyribonucleic Acid (rDNA) sequencing in Psomagen, United States of America (analyzing the obtained sequences in Basic Local Alignment Search Tool (https://blast.ncbi.nlm.nih.gov/Blast.cgi?PROGRAM=blastn&PAGE_TYPE=BlastSearch&LINK_LOC=blasthome, accessed on 2 February 2023)) and by the Biomerieux API-20e^MR^ standardized biochemical tests (20 tests made by triplicate for each strain) with comparison in the database of Biomerieux.

### 2.3. Microorganisms Selection for the Experiments in the Martian Regolith Analog

Two PGPB strains were selected, and one commercial formulation of AMF was chosen; the strains with all the plant benefit traits evaluated (with the internal codes V21AHO and C2A), and the commercial product BioMic TM-73^MR^ (which contains 20,000 viable spores of *Glomus fasciculatum*, *Glomus constrictum*, *Glomus tortuosum*, *Glomus geosporum*, *Acaulospora scrobiculata* and *Gigaspora margarita* per kilogram of product according to the provider).

### 2.4. Experiment Design for the Plant Growth Analysis

The cherry tomato (*Solanum lycopersicum* var. *cerasiforme*) was used as a study model for all the tests. The seeds, provided by local sellers, were disinfected with 0.5% chlorine, then washed with sterile milk, and finally washed with sterile water (to remove the fungicide from the seeds). Around 20 kg of the Martian Regolith Analog was sifted to 40 mesh and then placed in stainless steel cans and sterilized under dry heat in an oven (200 °C for 48 h) before use. Xinnun greenhouse seed germination containers measuring 6.3 cm × 18.3 cm × 14.1 cm were disinfected with concentrated chlorine and 70% (*v*/*v*) ethanol before use. Thirty grams of the Analog were placed in each container space (each container has a width of 3.8 cm and a length of 5.2 cm). To determine the effect of the AMF addition and bacterial addition on the plant growth in the Martian Regolith Analog, we followed this experimental design (Table 1):

**Table 1:** Experimental design.

	Treatment	Number of Plants
1	Control	12
2	LA ^1^	12
3	1% PC ^2^	12
4	1% PC and LA	12
5	AMF inoculation only ^3^	12
6	AMF inoculation and LA	12
7	1% PC and AMF inoculation	12
8	1% PC, AMF inoculation and LA	12
9	*V21AHO*	12
10	*V21AHO* with 1% PC	12
11	*V21AHO* with AMF inoculation	12
12	*V21AHO* with 1% PC and AMF inoculation	12
13	*C2A*	12
14	*C2A* with 1% PC	12
15	*C2A* with AMF inoculation	12
16	*C2A* with 1% PC and AMF inoculation	12
17	*C2A* and *V21AHO*	12
18	*C2A* and *V21AHO* with 1% PC	12
19	*C2A* and *V21AHO* with AMF inoculation	12
20	*C2A* and *V21AHO* with 1% PC and AMF inoculation	12

^1^ LA—Long–Ashton nutritive solution. ^2^ PC—Magnesium perchlorate in soil (mass percent). ^3^ AMF—Arbuscular Mycorrhizal Fungi Formulation (10% mass of substrate).

The seeds germinated in the containers over the analog, and then, the necessary seeds were selected to obtain exactly one plant per space; 240 were used in the experiment. Long–Ashton nutrient solution (KNO_3_ 0.404 g/L, MgSO_4_·7H_2_O 0.368 g/L, Ca(NO_3_)·4H_2_O 0.944 g/L, NaH_2_PO_4_·H_2_O 0.09862 g/L, MnSO_4_·H_2_O 0.00169 g/L, CuSO_4_·5H_2_O 0.00025 g/L, ZnSO_4_·7H_2_O 0.00029 g/L, H_3_BO_3_ 0.0031 g/L, NaCl 0.0059 g/L, (NH_4_)_6_Mo_7_O_24_·4H_2_O 0.000088 g/L, FeC_6_H_5_O_7_ 0.0249 g/L and H_3_C_6_H_5_O_7_·H_2_O 0.0249 g/L) was used to determine if nutrient availability is a significant factor for plant growth on the analog. Magnesium perchlorate was added to the analog at a concentration of 1% mass/mass (10,000 ppm), as reported by [20,21], which corresponds to the real Martian Regolith concentration. 3 mL of sterile water was used for irrigation every 3 days; meanwhile, in the plants supplemented with the Long–Ashton solution, 3 mL of the solution instead of water was used each week. The experiment lasted 45 days after germination, and all experiments were conducted in an incubation room maintained at 24 °C with a 16 h light (white cold light, fluorescent lamps, 16 Watts and 1600 lumens) period and an 8 h dark period. The plants for the bacterial treatments were inoculated with approximately 1 × 10^6^ cells at the beginning of each experiment. The inoculum was prepared in Trypticase Soy Broth; the broth was centrifuged to obtain a pellet. After that, the pellet was diluted and adjusted according to the absorbances obtained with a plate reader to the required optical density (approximately 0.1 OD600) that represents 10^6^ Colony-Forming Units according to the Agilent *E. coli* Cell Culture Concentration from Optical Density at 600 nm (OD600) Calculator (https://www.agilent.com/store/biocalculatorscalcODBacterial.jsp?_requestid=39274 (accessed on 4 June 2023)).

The fresh mass of the plant, shoot length, and root length were evaluated as growth parameters. The fresh mass of the total plants was determined using an analytical balance with a precision of 0.0001 g, and the root length and the shoot length were determined using a calibrator (vernier). The determination was made after 45 days.

To determine the AMF colonization in the roots of the selected experiments, we used the trypan blue method [22,23,24]. Plastic tubes containing the roots were flooded with a 10% Potassium Hydroxide (KOH) solution to clear the samples. They were placed in an electric autoclave for 10 min at 110 °C. After that, the roots were rinsed with tap water. Then, the roots were stained with 0.1% trypan blue dissolved in aceto-glycerol; subsequently, they were rinsed with distilled water and autoclaved again at 110 °C for an additional 10 min. The roots were stored in aceto-glycerol before being used. The roots were cut into 1 cm fragments to quantify colonization, then placed on slides in three blocks of 5 fragments (15 root pieces per plant) and analyzed directly in the microscope.

### 2.5. Statistical Analysis

To identify statistical differences among treatments, ANOVA test and Dunnett’s test were performed for all the growth parameters and root mycorrhizal colonization in all the treatments. The software used was MATLAB^MR^ R2023a using a significance level of 0.05 for all the analyses.

## 3. Results

### 3.1. Analog Selection and Characterization

**Figure 1:**
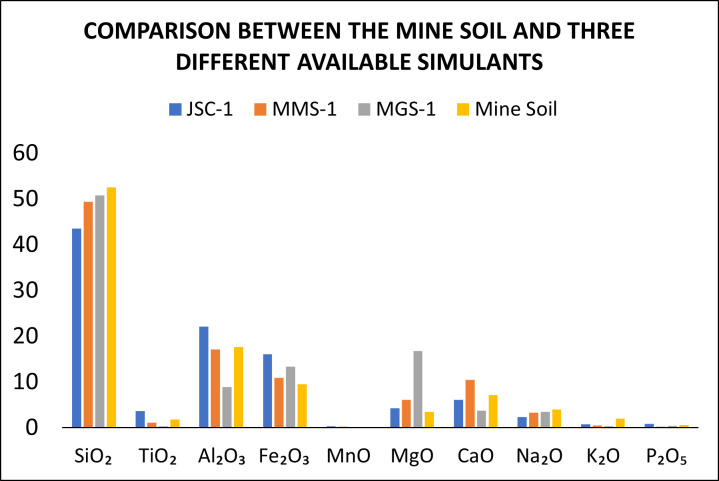
Mine soil chemical composition and comparison with other simulants [3] (a simulant is a material created on Earth designed to copy the physical and chemical properties of Space Bodies’ Regolith).

The mine soil had the following chemical composition (in mass percent): 52.54% of SiO_2_, 1.81% TiO_2_, 17.66% of Al_2_O_3_, 9.46% of Fe_2_O_3_, 0.145% of MnO, 3.43% of MgO, 7.09% of CaO, 3.95% of Na_2_O, 1.96% of K_2_O, and 0.55% of P_2_O_5_ according to X-ray fluorescence. The results of the XRF analysis are summarized in the following graphic, which compares them with other Martian Regolith Simulants (Figure 1).

The average pH of the analog was 5.869 ± 0.226; this value is lower than the Martian Regolith’s pH of 7.2 reported by Plumb, R. C., Bishop, J. L., & Edwards, J. O. [25] from Viking probe data, estimated from a mathematical model based on the formation of metallic carbonates and the dissociation constants of soluble acids present in a 0.5 mL of Regolith. The Martian Regolith pH is higher due to the presence of carbonates and nitrates, but this is not a bad value at all, as plants like tomatoes can grow at those pH levels, and good data can be obtained using different stressors (such as heavy metals and perchlorates).

The sample yields 0.945% ± 0.17% of total organic matter. That is an excellent value because, with the data recovered at this moment, there is not a significant amount of organic matter in the Martian Regolith [21], so, by using this analog, the supplementation of organic matter can be tested to determine the effect of this parameter on the plant’s growth in future research.

### 3.2. Perchlorate Tolerant Microorganisms Isolated and Their Plant-Beneficial Traits

Fourteen different strains were obtained from the soils used in this research, 12 from the mine soil and two from the arable soil (*). The internal codes used for the strains are as follows: *C2A, M1A1B, M1A2B, M2A1P, MIB1H, MIB2B, *P1A, V11AB, V11AP, V11AR, V21AHO, V22BR, V31BB, and V32AO. In Table 2, we summarize the plant’s beneficial traits identified in them.

According to the rRNA 16S sequencing and API-20E tests, the strains *C2A, *P1A, and M1B1H are species of *Enterobacter cloacae*, and the strain V21AHO is a *Klebsiella oxytoca* specie. The data from Psomagen sequencing are available in the GenBank repositories OR607676, OR607677, OR607678, and OR607679. Presented in Table 3 are the results of the API 20E tests performed on the selected strains, which served as the confirmation criteria for strain identification.

As a final test before beginning the experiments on the analog, we conducted a quick inoculation test using *Arabidopsis thaliana* plates with Murashige–Skoog agar to determine whether the selected bacteria would negatively affect plant growth. The results are summarized in Table 4.

The strains *P1A and M1B1H inhibit the growth of *Arabidopsis thaliana*; therefore, they are not suitable as biofertilizers. Further research can confirm whether these strains are harmful to other plants or only to *Arabidopsis thaliana*. Meanwhile, both strains were discarded from experiments with the analog, and only *Enterobacter cloacae* C2A and *Klebsiella oxytoca* V21AHO were used.

### 3.3. Growth Parameters Determination and Statistical Analysis

**Figure 2:**
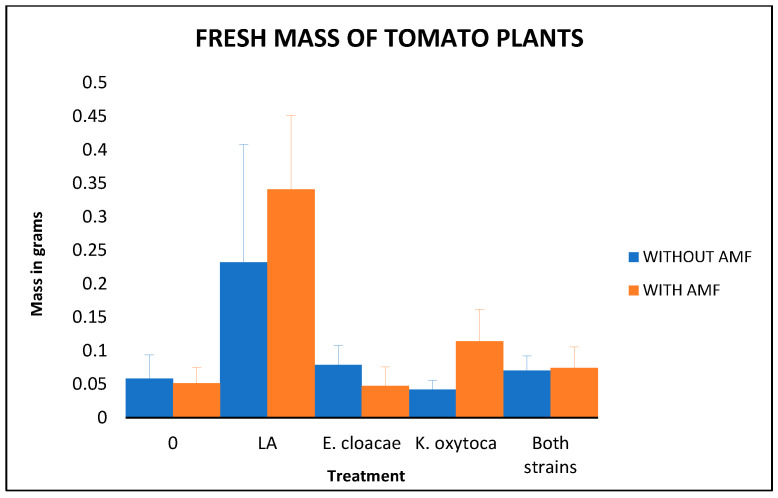
Fresh mass of the tomato plants’ growth over the analog in grams: LA means the supplementation with Long–Ashton solution and AMF means the inoculation with Arbuscular Mycorrhizal Fungi. Bars indicate standard deviation.

The results obtained by the plant measures showed that the perchlorate in the soil at 1.0% concentration was lethal to tomato plants, because all the 120 plants died in less than 2 weeks after being transplanted in the polluted analog; on the other hand, all the plants transplanted in the analog without perchlorate grew without problems, having a viability of the 100%. These results are similar to those reported by [1,2] using different plants and by [5] using *Arabidopsis* and lettuce. First, we present the fresh mass results, including a graphic comparison (Figure 2) and the ANOVA table (Table 5).

As we can see, there are significant differences between the treatments. To determine differences from the control plants, we used a Dunnett test. The results show that both Long–Ashton treatments exhibit the most growth; the *p*-value for the Long–Ashton treatment with AMF was 0, and the *p*-value for the Long–Ashton treatment without AMF was 1.5652 × 10^−7^.

**Figure 3:**
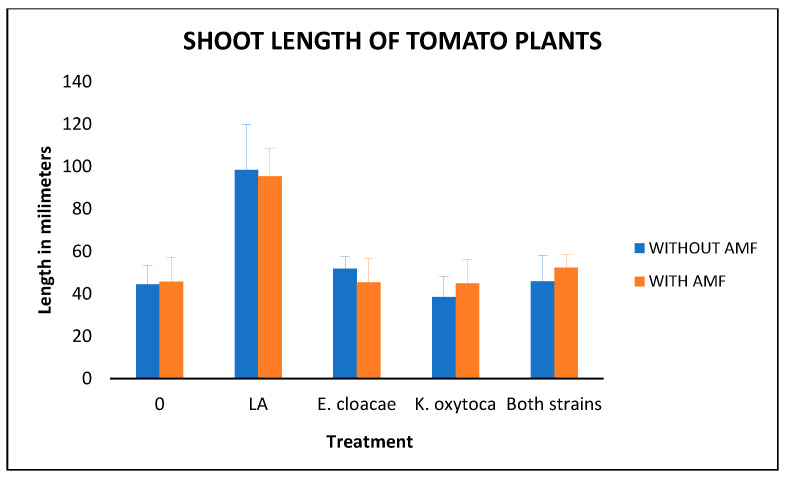
Shoot length of the tomato plants’ growth over the analog in millimeters: LA means the supplementation with Long–Ashton solution and AMF means the inoculation with Arbuscular Mycorrhizal Fungi. Bars indicate standard deviation.

Second, we present the shoot length results, including a graphic comparison (Figure 3) and the ANOVA test (Table 6).

As we can see, there are significant differences between the treatments. To determine differences from the control plants, we used the Dunnett test. The results again show that the Long–Ashton treatments have the most growth; the *p*-value for the Long–Ashton treatment with AMF was 0, and that for the Long–Ashton treatment without AMF was also 0.

**Figure 4:**
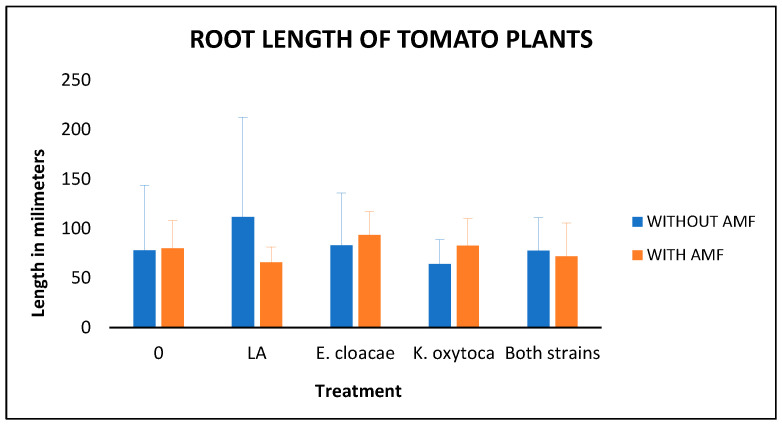
Root length of the tomato plants’ growth over the analog in millimeters: LA means the supplementation with Long–Ashton solution and AMF means the inoculation with Arbuscular Mycorrhizal Fungi. Bars indicate standard deviation.

Finally, we present the root length results, including a graphical comparison (Figure 4) and the ANOVA test (Table 7). In this parameter, there were no significant differences among treatments, suggesting that container size influenced root development.

The results of analyzing the growth parameters are like those reported by [5], who found that the problem is not the chemical composition of the simulants and analogs but rather their nutrient availability.

### 3.4. Estimation of Arbuscular Mycorrhizal Fungi Colonization in Roots

**Figure 5:**
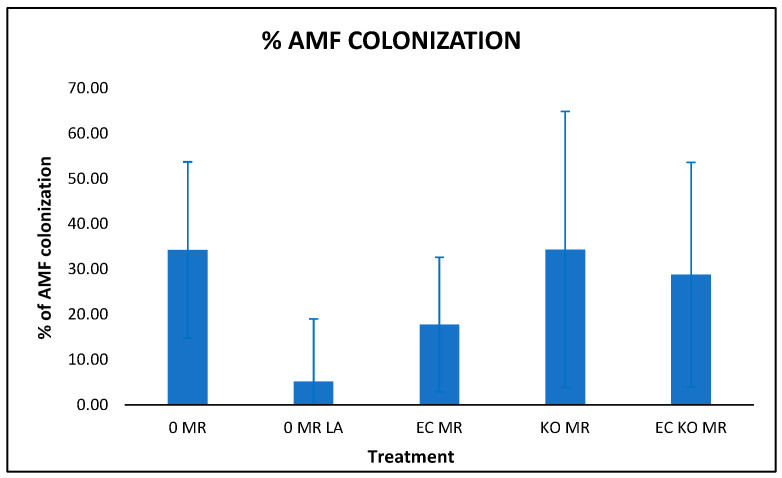
Percemtage of AMF colonization in the roots of the tomato plants: MR indicates the Mycorrhizal inoculation, LA means Long–Ashton supplementation, EC means inoculation with *Enterobacter cloacae* and KO means inoculation with *Klebsiella oxytoca*. Bars indicate standard deviation.

The percentages of Arbuscular Mycorrhizal Fungi colonization are shown in Figure 5, with the ANOVA results summarized in Table 8.

According to the results, there are significant differences between the treatments; a Dunnett test was performed to determine differences relative to the control, but only the Long–Ashton solution showed a significant difference.

## 4. Discussion

### 4.1. Analog Properties

As can be seen, the principal differences between the different analogs are the total Fe_2_O_3_ content, the SiO_2_ content, and the K_2_O content. The Fe_2_O_3_ content is lower than that of the Johnson Space Center Number One (JSC-1), Mojave Mars Simulant Number One (MMS-1), and Mars Global Simulant Number 1 (MGS-1), according to the data provided by [3]. The principal advantages of our analog are its price and availability, as the other three simulants are expensive and hard to obtain (https://www.themartiangarden.com (accessed on 5 February 2021)). In this context, the mine soil can be a valuable resource for research in Mexico, particularly in laboratories with budget constraints. The analog can be recollected, mixed, and milled using standard equipment, such as mechanical mixers and sieves. Further research can be made to determine their physical properties and their mineral structure and it may be worth studying whether the heavy metal content of the analog is a critical factor in plant development and interactions with microorganisms.

### 4.2. Isolated Microorganisms Properties

Some *Klebsiella* and *Enterobacter* strains were reported in the past as Mycorrhizal Helper Bacteria [26], and, in a more specific way, *Klebsiella* was studied before as a potential biofertilizer in Moon Regolith Simulants [6]; further studies using simulants and Arbuscular Mycorrhizal Fungi (AMF) are suggested to establish the true potential of these two strains in different simulants and analogs. *Klebsiella oxytoca* is an opportunistic pathogen in humans but is considered a bacterium of biotechnological importance. It belongs to the *Proteobacteria* phylum, the *Gamma proteobacteria* class, and the *Enterobacteriaceae* family. It is a Gram-negative, non-motile, rod-shaped bacterium that is found in natural environments. Its importance varies because it can be used to produce chemical compounds, such as biofuels (ethanol and hydrogen), fix nitrogen, and facilitate bioremediation [27]. *Enterobacter cloacae* is a Gram-negative bacillus that is naturally distributed in many environments; it is considered an opportunistic pathogen that is present in hospital-acquired infections (around 5%) that can cause pneumonia, meningitis, septicemia, cerebral abscess, and urinal and intestinal infections [28]; on the other hand, *Enterobacter cloacae* is considered a Plant Growth-Promoting Bacteria because it promotes the production of Indole Acetic Acid, increases the plant’s growth, and increases the curcuminoid production in selected plants [29,30]. Both microorganisms can be pathogens, but they are also promising alternatives for chemical production and as effective biofertilizers, and they can act as endophytes in some plants. Therefore, instead of considering their possible pathogenicity, they may be good alternatives for long-term sustainability [29]. At this moment, there is little information about the perchlorate tolerance of *Klebsiella oxytoca* and *Enterobacter cloacae*, so we can say that could be interesting to study if there are a health risks associated with those bacteria. And if those strains are safe to humans, whether they can be used as biofertilizers should be explored in future studies, because those bacteria have interesting potential from the point of view of agronomists [27,28,29,30], due to its solubilization of phosphate and potassium, its siderophore production and its nitrogen fixation capabilities. Additionally, in vitro tests using *E. cloacae* and *K. oxytoca* are recommended to identify and quantify their possible perchlorate degradation capabilities. That is because, as of June 2025, the UniProt database does not have records of the presence of perchlorate reductase and Chlorite dismutase (two key enzymes in perchlorate metabolism) in both organisms. Perchlorates are a common contaminant in areas with explosive manufacturing industries, so research into their biological degradation can benefit humans.

### 4.3. Plant Growth Parameters and AMF Data

At least under the conditions used, the chemical composition of the analog is not a problem for tomato plant growth, but perchlorate itself is the principal threat. Our results are similar to those obtained by [5] in 2021. In their tests using the Hoagland solution, they obtained growth in *Arabidopsis* for 10 days at a high viability rate. Additionally, these results differ from those reported by [31], as the Martian Regolith Analog did not inhibit tomato growth under the conditions used in these experiments. Hence, chemical composition is a lesser problem than the perchlorate toxicity. Our conclusion is like that determined by [1,2]: plants can grow in the analogs without problems, and if nutrient availability exists, crops can be cultivated. Further studies with different crops can be conducted to determine their tolerance to perchlorate.

The AMF inoculation has a low value in Long–Ashton-supplemented plants. That is an expected result because the nutritive solution provides sufficient phosphorus to the plants, and AMF colonization is inhibited by the phosphorus abundance [32]. We can say that there was good colonization because, in three of the other treatments, the percentage was higher than 25% in 45 days; this colonization value in tomato is higher than the one obtained in 35 days by [33], indicating that the mine soil used in the experiments does not inhibit the development of the AMF. An interesting factor is that *Klebsiella oxytoca* and *Enterobacter cloacae* are compatible with the AMF formulation; therefore, studying their interactions is a viable research area in the fields of biofertilizers and bio-stimulants. According to the obtained data, the use of PGPB and AMF did not improve the growth of tomato plants compared to the analog over the 45 days recorded, so further research is recommended to determine whether a different effect is observed in other study models (such as lettuce), as reported by [10].

The use of Arbuscular Mycorrhizal Fungi in Martian Regolith Analogs could be very important for future Martian agriculture, because on Mars, there will not be short-term infrastructure to produce fertilizers, so we will need to survive with the planet’s available resources. The AMF helps plants in many ways [23,24], so the discovery of its role in plant growth in analogs can provide a different perspective. For example, the Moon Regolith does not contain perchlorate, but it does contain heavy metals like iron and aluminum, so the discoveries we made here can help research to establish Moon agriculture using microorganisms. It will be easier and more economical to transport packages of preserved microorganisms than to transport packages of chemical biofertilizers into space.

## 5. Conclusions

The mine soil showed a good chemical composition compared with other available Martian Regolith Simulants and Analogs, making it an economical alternative for use in Mexico. The four selected strains solubilize phosphate and potassium, produce siderophores, and fix nitrogen, making them potential biofertilizers. The analog itself does not affect tomato growth, but perchlorate at 10,000 ppm inhibits plant development. There is mycorrhizal colonization in all treatments, so we can say that the iron and aluminum content in the analog used does not harm the AMF. The bacterial and AMF inoculations did not improve tomato development in the experiments because the treatments did not show statistical differences.

According to the previous statements, we can say that research guided towards increasing the nutrient availability in the Martian Regolith will be crucial in future studies because, in the scenario where there is existing technology to remove the perchlorate from the Martian Regolith, synthetic fertilizers will not be available, so is essential to study alternative ways to obtain nutrients from the mineralized forms that exist in the Martian Regolith itself.

## Figures and Tables

**Table 2:** Summary of the plant’s beneficial traits identified in the isolated microorganisms.

Strain	Phosphorous Solubilization	Potassium Solubilization	Siderophore Production	Nitrogen Fixation
*C2A	✓	✓	✓	✓
**M1A1B**	✓	X	X	X
**M1A2B**	X	X	X	X
**M2A1P**	X	X	X	X
**MIB1H**	✓	✓	✓	✓
**MIB2B**	✓	X	X	X
*P1A	✓	✓	✓	✓
**V11AB**	X	X	X	✓
**V11AP**	X	X	X	✓
**V11AR**	X	X	X	✓
**V21AHO**	✓	✓	✓	✓
**V22BR**	X	X	X	X
**V31BB**	X	X	X	X
**V32AO**	✓	X	X	✓

✓ indicates a positive result in the biochemical test and X indicates a negative in the biochemical test.

**Table 3:** Results of the API20e tests in the selected microorganisms.

Test Strain	ONPG	ADH	LDC	ODC	LCIT J	H_2_S	URE	TDA	IND	VP	GEL	GLU	MAN	INO	SOR	RHA	SAC	MEL	AMY	ARA
**Control**	−	−	−	−	−	−	−	−	−	−	−	−	−	−	−	−	−	−	−	−
*C2A	+	+	−	+	+	−	−	−	−	+	−	+	+	−	+	+	+	−	+	+
**MIB1H**	+	+	−	+	+	−	−	−	−	+	−	+	+	+	+	+	+	+	+	+
*P1A	+	+	−	+	+	−	−	−	−	+	−	+	+	−	+	+	+	+	+	+
**V21AHO**	−	−	+	−	+	−	+	−	+	+	−	+	+	+	+	+	+	+	+	+

+ indicates a positive result in the biochemical test and − indicates a negative result in the biochemical test.

**Table 4:** Results of the microorganisms’ inoculation in Arabidopsis growth.

Strain	*Arabidopsis* Growth
**Control**	✓
*C2A	✓
**MIB1H**	X
*P1A	X
**V21AHO**	✓

✓ indicates that the strain does not show inhibition of the *Arabidopsis* growth and X indicates the opposite.

**Table 5:** Fresh mass ANOVA.

Source	SS	df	MS	F	Prob > F
Columns	1.03981	9	0.11553	22.93	1.65861 × 10^−21^
Error	0.55433	110	0.00504		
Total	1.59414	119			

SS means Sum of Squares, df means degrees of freedom, MS means Mean Square, F means F-test value and Prob > F means the probability.

**Table 6:** Shoot length ANOVA.

Source	SS	df	MS	F	Prob > F
Columns	50,992.3	9	5665.81	40.03	9.67681 × 10^−31^
Error	15,568.1	110	141.53		
Total	66,560.3	119			

**Table 7:** Root length ANOVA.

Source	SS	df	MS	F	Prob > F
Columns	20,844.3	9	2316.03	1.03	0.4211
Error	247,391.9	110	2249.02		
Total	268,236.1	119			

**Table 8:** AMF colonization ANOVA.

Source	SS	df	MS	F	Prob > F
Columns	0.76056	4	0.19014	4.06	0.0059
Error	2.57682	55	0.04685		
Total	3.33737	59			

## Data Availability

The data presented in this study are available in GENBANK at https://www.ncbi.nlm.nih.gov/genbank/ (accessed on 21 July 2025), reference number OR607676, OR607677, OR607678 and OR607679.
